# The effect of rhythm training on the motor coordination abilities of 8–12-year-old freestyle swimmers

**DOI:** 10.7717/peerj.15667

**Published:** 2023-07-28

**Authors:** Xiaofeng Yin, Rongxin Zhu, Xin Shi, Guang Cai, Chen Jing, Qile Pan, Tao Yang

**Affiliations:** 1 Department of Talent Identification and Development in Sport, Shanghai Research Institute of Sports Science (Shanghai Anti-doping Agency), Shanghai, China; 2 Xianda College of Economics and Humanities, Shanghai International Studies University, Shanghai, China; 3 School of P.E and Sport Training, Shanghai University of Sport, Shanghai, China

**Keywords:** Young athletes, Early coordination development, General coordination, Specific coordination

## Abstract

This study aims to evaluate the effect of rhythm training in the early coordination development and to find out its role in the transfer of specific performance in young swimmers. Eighty young swimmers aged eight to twelve were divided randomly into a control group (CON, *n* = 40, M/F=17/23) or an experimental group (EXP, *n* = 40, M/F=19/21). A total of 72 swimmers completed all the training programs and tests included in this study (CON, *n* = 35, M/F=15/20; EXP, *n* = 37, M/F=16/21). The training programme lasted 12 weeks. During the study period, all groups participated in the same swimming training program with the control group participating six times a week, and the experimental group participating four times a week with an additional two rhythm trainings each week. The height, weight, walking backwards ability, jumping sideways ability, and 25 m freestyle were measured for each participant and an index of coordination was analyzed from recordings of the participant’s 25 m freestyle. The results indicated that participation in rhythm training resulted in improved general motor and swimming-specific coordination among the swimmers, but the results varied by number of years of sport-specific training experience. The swimmers with more training experience improved more in swimming-specific coordination but less in general motor coordination. This study strengthens the evidence for the effectiveness of early rhythm training in swimmers, indicating that it is feasible to design programs to address general and sport-specific coordination in young athletes.

## Introduction

Motor coordination is the ability to coordinate all parts of the body in time and space during exercise to complete movements in a rational and effective way (Zahradník & Korvas, 2017). Motor coordination could be considered the external manifestation of the central nervous system’s level of control and regulation of the body’s movements.

Unlike other physical qualities that depend primarily on the body’s energy supply system, motor coordination is mainly determined by motor control and regulation. Good coordination allows individuals to complete movements safely and efficiently in various situations and motor coordination is the basis for quickly learning and mastering the physical movements associated with specific sports (Weineck et al., 2012).

Sports scientists have recently begun to study the impact of motor coordination among children and adolescents (Jaakkola, Watt & Kalaja, 2017), and have found that motor coordination is a strong predictor of athletic success in young athletes (Pion et al., 2015; Vandorpe et al., 2012; Waldziński, 2013). Although previous studies on the topic used differing research methods, the results indicate that the greatest adaptability of motor coordinative abilities occurs between 7 and 12 years of age, which is when the motor centers in the brain are developing (Gierczuk & Sadowski, 2015; Golle, Mechling & Granacher, 2019; Weineck, 1996). During this period, coordination abilities have developed to 75% of their maximum level (Lyakh, Jaworski & Wieczorek, 2007). The onset of adolescent growth spurt, hormonal changes and rapid developments in the skeletal muscle system may lead to difficulties executing whole-body movements, affecting coordination performance in complex environments or stressful conditions (Kröger, Roth & Schmidt, 2014). Because of this, it might be more efficient to focus on coordination development before puberty.

Due to the high plasticity and adaptability of the brain and its rapid development in childhood, this phase offers particularly favorable conditions for the optimization of coordinative abilities. At no other point in time can the motor cortex—the area of the cerebral cortex responsible for the execution of all motor actions—be trained better than in childhood and adolescence, so it is important to make optimal use of this learning age (Neuper, 2015; Weineck et al., 2012).

The development of motor coordinative abilities is crucial in all cyclic sports, especially swimming. Because water is about 1,000 times denser than air, the resistance generated in water requires specific movement patterns to overcome. Swimmers must balance using rhythmic movements of the limbs to produce the best propulsion effect while trying to keep the resistance caused by stroking to a minimum (Frank, 2008).

Every sport has a different time series distribution of movement, but excellent motor rhythm ability is required for all smooth, fluid movements (Miura et al., 2011). Rhythm training (RT) combines beats with the movement and coordination of the upper and lower limbs, varying from simple to complex movements and from slow to fast tempos. RT helps establish good proprioception ability and improves motor coordination ability.

The trainability and transferability of motor coordination is still unknown, despite several studies on the topic (Jagiełlo & Kalina, 2007; Jerzy et al., 2015; Vandorpe et al., 2012). However, researchers have found that intra- and inter-limb motor coupling can be achieved by adjusting environmental, organismal and task-related constraint variables to influence front crawl stroke performance and specific coordination performance in young swimmers (Figueiredo et al., 2016; Silva et al., 2019a; Silva et al., 2022). Therefore, practitioners should consider designing a representative learning situation that allows young swimmers to create paths for spontaneous exploration of available motor solutions, rather than always pursuing ideal or optimal coordination models (Dekerle, 2020).

Motor coordination is considered to be related to age and remains relatively stable during childhood (Magill & Anderson, 2010). Greater variability in motor coordination was found in older children over time. Vandorpe et al. (2012) verified the stability of motor coordination in childhood. Their study observed no sign of motor coordination development in children after three years of active participation in sports clubs. Another study also found high stability in motor coordination over two years in elementary school students, with a stability coefficient exceeding 0.70 (Ahnert, Schneider & Bös, 2010). However, there is also opposing evidence that suggests motor coordination may not be stable, and that active participation in specific movements is associated with the specific development of motor coordination. Opstoel et al. (2015) found that motor coordination in young athletes playing different sports is more varied than in previous, similar studies. For example, motor coordination differences found in gymnastics, swimming, and hockey athletes may be related to the athlete’s history and intensity of sport-specific training in those sports (Jaakkola, Watt & Kalaja, 2017).

Although general coordination is known to have a positive effect on any sport-specific training, a direct connection between the development of general coordination and improvement in highly specialized athletic techniques has not yet been established (Nieber, 2015). This seems to suggest that general motor coordination abilities need to be developed before starting structured training and should be done early in the training of adolescent athletes.

This study selected young swimmers in the critical development period of coordination ability as the experimental subjects because specific upper limb coordination patterns in swimming are strongly correlated with swimming speed and stroke frequency range (Seifert et al., 2014; Silva et al., 2019a; Silva et al., 2019b). Young swimmers need both excellent upper-limb coordination and an adaptive behavioral flexibility. This study aims to investigate the effect of general coordination training represented by RT in early coordination development, and the role of general coordination training in the transfer of specific performance of young swimmers. This study specifically addresses the following research questions: (1) Does RT have an impact on general and sport-specific coordination in young swimmers with different specific training experience? (2) Does RT have a direct impact on sport-specific performance improvements in young swimmers?

## Materials & Methods

### Participants and design

A sample of 90 swimmers aged between 8 and 12 years volunteered to take part in this study. Only 80 athletes completed the full training intervention (10 swimmers were excluded from the study due to absenteeism). All swimmers from the same youth swimming school were randomly assigned to either the experimental group (EXP, *n* = 40, M/F =17/23) or the control group (CON, *n* = 40, M/F =19/21) according to training experience: E1 *vs* C1 (aged 7.74 ± 0.23 *vs.* 7.49 ± 0.82), E2 *vs* C2 (aged 8.87 ± 0.21 *vs.* 8.75 ± 0.27), and E3 *vs* C3 (aged 10.78 ± 0.33 *vs.* 10.66 ± 0.16). A further eight swimmers were excluded for missing one or more testing sessions, leaving a total of 72 participants. Table 1 shows the participant characteristics of the EXP (*n* = 37, *M*/*F* = 16/21) and CON (*n* = 35, M/F =15/20) groups. Table 2 shows the performance of the participants in baseline testing. Coaches evaluated athletes based on their level of swimming ability and by the swimming competitions in which they competed. All participants were at a similar entry level. Both groups of participants were in a competition period (2–3 competitions in 12 weeks). No participants had previous formal strength and conditioning training experience.

**Table 1:** Anthropometric characteristics of study participants. Values are reported as means ± standard deviations (SD).

	trained one year		trained two years		trained four years	
	C1 (*n* = 18)	E1 (*n* = 18)	C2 (*n* = 6)	E2 (*n* = 11)	C3 (*n* = 11)	E3 (*n* = 8)
Gender(M/F)	7/11	6/12	3/3	6/5	5/6	4/4
Age (Yr)	7.74 ± 0.23	7.49 ± 0.82	8.87 ± 0.21	8.75 ± 0.27	10.78 ± 0.33	10.66 ± 0.16
Height (cm)	134.00 ± 6.21	131.32 ± 5.42	138.77 ± 4.91	138.71 ± 5.66	148.02 ± 3.9	149.61 ± 4.19
Weight (kg)	28.84 ± 5.49	27.51 ± 5.39	32.80 ± 7.51	31.29 ± 5.99	39.21 ± 5.66	38.04 ± 3.61
BMI (kg/m^2^)	15.97 ± 2.06	15.86 ± 2.24	16.92 ± 3.12	16.14 ± 2.11	17.84 ± 1.94	17.01 ± 1.61

**Notes.**

Values are reported as means ± standard deviations (SD)

E experimental group C control group BMI body mass index

**Table 2:** Coordination characteristics of study participants.

	trained one year				trained two years				trained four years			
	C1 (*n* = 18)	CV (%)	E1 (*n* = 18)	CV (%)	C2 (*n* = 6)	CV (%)	E2 (*n* = 11)	CV (%)	C3 (*n* = 11)	CV (%)	E3 (*n* = 8)	CV (%)
Walking Backwards	28.56 ± 9.50	33.26	35.61 ± 9.20^*^	25.84	29.00 ± 7.87	27.14	36.45 ± 5.65^*^	15.5	32.73 ± 8.92	27.25	31.00 ± 7.91	25.52
Jumping Sideways	31.67 ± 5.01	15.82	34.33 ± 5.37	15.64	36.67 ± 6.28	17.13	44.09 ± 5.99^*^	13.59	37.05 ± 2.36	6.37	39.25 ± 4.64	11.82
25m Freestyle	20.18 ± 1.49	7.38	18.80 ± 1.50^*^	7.98	17.67 ± 1.40	7.92	16.63 ± 1.05	6.31	15.34 ± 0.57	3.72	14.20 ± 0.50^*^	3.52
IDC	0.13 ± 0.07	53.85	0.15 ± 0.05	33.33	0.07 ± 0.02	28.57	0.09 ± 0.02	22.22	0.12 ± 0.03	25	0.13 ± 0.03	23.08

**Notes.**

Values are reported as means ± standard deviations (SD)

E experimental group C control group BMI body mass index CV Coefficient of Variation

* EXP swimmers are significantly different from CON swimmers with the same number of training years (*p* < 0.05).

The inclusion criteria for participants were as follows: (1) Healthy swimmers with no history of injury during sporting activities (training and/or competition). (2) Swimmers who trained an average of 2-hours/day from Monday to Saturday, for a total of 12 h per week, and often participated in competitions. (3) Swimmers who did not participate in formalized training for other sports.

The exclusion criteria for participants were as follows: (1) Recreational or untrained swimmers. (2) Swimmers with less than five training sessions per week. (3) Swimmers with a history of muscle or joint injuries. (4) Swimmers with poor training attendance rates (below 80%). (5) Swimmers who were involved in a training program for another sport.

This study was approved by the Shanghai Research Institute of Sport Science Ethics Committee (IRB approval number: LLSC20210004). Written, informed parental consent as well as participant assent was collected for each study participant.

### Procedures

**Figure 1:**
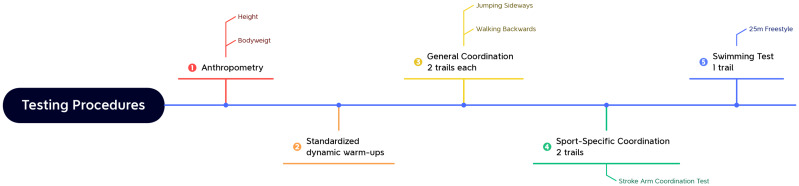
Testing procedures.

During the 12-week intervention period, the CON group continued with their regular trainings, including 35 min of swimming-specific training, such as on-land skills simulations. The EXP group replaced two of their regular training sessions each week with 35-minute RT sessions. Study participants received no other formal conditioning. All participants were tested using identical methods by the same testers both the week before and the week after the 12-week program. All tests were performed at the same swim school where the participants trained. Swimmers performed general coordination tests (including walking backwards and jumping sideways) wearing comfortable sneakers and sports clothes, while the sport-specific coordination tests (including the stroke arm coordination test) were completed in the swimming pool. The testing procedures are shown in Fig. 1. First, the heights and weights of the swimmers were measured. Then, the swimmers performed a standardized 10-minute dynamic warm up and practice trials before the formal tests. After the warm-ups, swimmers were tested in the following order for the test battery: jumping sideways, walking backwards, stroke arm coordination test, and 25 m freestyle performance. Jumping sideways and walking backwards were subtests of the Deutscher Motorik-Test 6–18 (Bös & Schlenker, 2011), which has been shown to be reliable (Kiphard & Schilling, 2007). The swimmers performed two trials of the jumping sideways (15 s), walking backwards, and stroke arm coordination tests, along with one 25 m freestyle performance trial, based on previously reported guidelines. Participants had a 60-second rest period between trials and a 5-minute break between each test to minimize the effect of fatigue on coordination variables.

**Anthropometry.** Height (H) was measured to the nearest 0.1 cm (Height Tester, Shanghai Quyi Technology Company, Shanghai, China), body weight (BW) was measured to the nearest 0.1 kg (HD-394; Tanita, Tokyo, Japan) following manufacturer guidelines and using previously published, standardized methods (Lohman, Roche & Martorell, 1988). Body Mass Index (BMI) was calculated using formula as follow, BMI = weight (kg)/[height (m)]^2^.

**General coordination.** General coordination was evaluated by two subtests of the Deutscher Motorik-Test 6–18 (DMT6-18; Bös & Schlenker, 2011). These two subtests, the jumping sideways test and the walking backwards test, provided an assessment of the speed of movement coordination and precision of movement coordination of the participants, respectively. For the jumping sideways (JS) test, the participants were asked to jump sideways, with both legs together, as many times as possible in 15s over the center line of a rectangular area (50 × 100 cm). The number of correct jumps from the two trials were added together as the final raw result. This test is reliable (test-retest reliability = 0.96, *P* < 0.00) as shown in previous studies (Bös & Schlenker, 2011; Oberger et al., 2006). For the walking backwards (WB) test, subjects were asked to walk backwards three times on each of three beams: (a) a 3-meter beam with a width of six cm; (b) a 3-meter beam with a width of 4.5 cm; and (c) a 3-meter beam with a width of three cm. Total steps taken before falling from the beam were taken as the results of each trail. The maximum score of each trail was eight steps. Other researchers have examined the test-retest reliability of WB, with an intraclass correlation coefficient of 0.80 (*p* < 0.05; Kiphard & Schilling, 2007). Moreover, the results of the JS test and the walking backwards test can also discriminate between performance levels (Bös & Schlenker, 2011; Platvoet et al., 2018); (Vandorpe et al., 2011).

**Figure 2:**
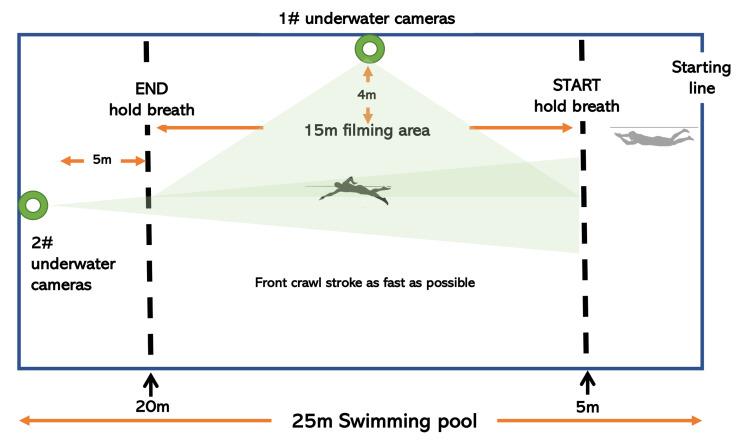
Camera setups.

**Sport-specific coordination.** Stroke phase as well as arm coordination during front crawl swimming were quantified using the index of coordination (IdC), which describes coordination patterns by measuring the lag time (LT) between the propulsion phase of each arm (Chollet, Chalies & Chatard, 2000). Swimmers started in the water against the wall of the pool. Each swimmer performed two swim trials of swimming the front crawl stroke as fast as possible for 15 m in a 25-meter pool, while holding their breath to avoid modifications in coordination. Before entering the recording zone, the swimmers did not stroke but glided through the water. The first four stroke cycles of each swimmer’s right arm were recorded from the time they entered the 5–20 meter camera filming area for further IdC analysis. The swimmers rested for at least 2.5 min between each trail, and the participants were informed of their performance results at the end of each trail. Two cameras (GoPro 5) set with high shutter speeds were used to capture the stroke phases as well as modes of arm coordination underwater. All videos were recorded at 25 frames per second. One camera recorded the swimmer from the front, and the other camera was placed close to the inner wall of the pool to capture the profile of the swimmer (Fig. 2). The Dartfish motion video analysis software was used to analyze the coordinated pattern of the participants in four cycles of the freestyle arm stroke, as recorded from 5 m to 20 m, and then a mean IdC was calculated. The equation (E3) for calculating the IdC can be found in Article S1. To better identify coordination changes, instead of labeling the IdC positive or negative based on the coordination pattern of the arm, we used the absolute value of the IdC in this study to assess changes in coordination.

### Training program

**Figure 3:**
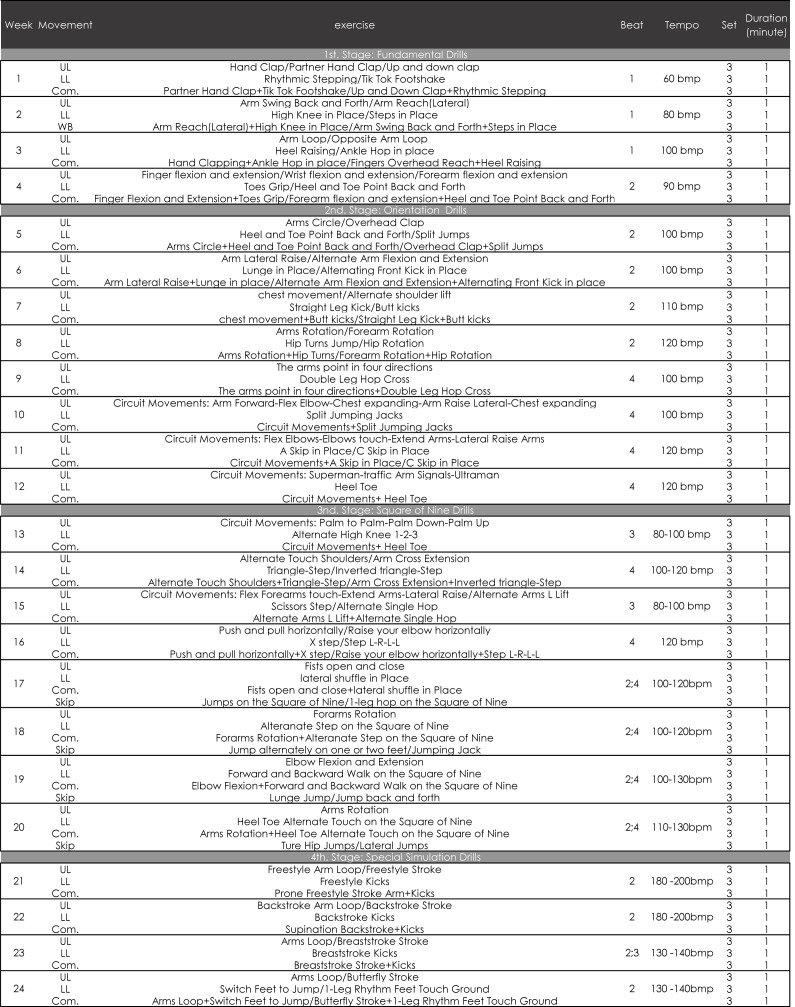
Rhythm training program structure.

A certified strength and conditioning specialist supervised the training program to ensure accurate technical execution throughout the entire study. The training program used in this study was designed using a step-wise RT approach based on training programs that have previously been shown to be effective in young athletes (Aly Shady & Abdelsattar Mahmoud, 2014; Söğüt, Kirazci & Korkusuz, 2012). The program included four phases based on coordination requirements and tempo rates (Fig. 3). The RT training was done out of the water, but the training included both in-phase and anti-phase movement patterns, some of which are consistent with the needs of swimming-specific movements. In the first phase of two weeks, also known as the *Adaptation Phase*, the goal was to establish a basic sense of rhythm in the participants. In the second period, or the *Learning Phase* (weeks 3–6), participants learned and mastered more types of rhythm-based exercises. During the third period, or the *Intensive Phase* (weeks 7–10), the perceptions and nervous systems of the participants were stimulated as they were asked to perform unfamiliar movements at different tempos. This phase included more difficult exercises, including asymmetrical movement patterns. The main goal of the final *Transition Period* (11–12 weeks) was to allow participants to combine the type of rhythm they had learned with swimming-specific techniques, thereby optimizing their sport-specific coordination.

Each session included three parts: upper-limb exercises (approximately 10-min), lower-limb exercises (approximately 8-min), and combination exercises (approximately 8-min). Details of the training program are shown in Fig. 3. A standard metronome (version 3.30 in the app store of iPhone iOS, developed by Gismart Limited) was used to set the tempos for all training sessions. Rhythmic movements were synchronized with the beats of the metronome and practiced by the EXP group for 12 weeks. The rhythm training was performed before the swim training twice a week for 35 min each time.

### Data collection

Data were collected from September 2021 to December 2021. For each swimmer, the motor coordination test took about 10 min and the swim coordination test took about five minutes. Well-trained physical education students who were blinded to all pre-test results, conducted the tests. The gender, date of birth and class of the swimmer were all recorded in addition to the test results. At the baseline measurement (T0), participants were also asked if they participated in formal training for other sports (yes/no).

### Statistical analyses

Data were presented as mean ± standard deviation (SD). An independent sample T test was used to compare the differences between CON and EXP swimmers with the same number of training years pre or post training. Effect sizes (ES) were determined by Cohen’s d and were classified as “trivial”, “small”, “medium”, “large” or “very large” (Hopkins, 2002). A within group ES (calculated by: (Post-Pre)/SD, with SD here being the SDs of same-gender individuals within the group) was used to examine the individual response to the rhythm training. A Levene’s test was used to determine the homogeneity of variance across the different groups. If all variances were equal, the difference of within group ES between the groups was analyzed using a two (groups: CON, EXP) by three (training years: 1, 2, 4) analysis of variance. If the interactions of group × training years was significant, a simple contrast analysis was used. One way ANOVA was used for a simple contrast analysis, and a post-hoc Scheffe test was used to compare the differences between the different numbers of training years. If all variances were not equal (changes of IdC), an independent sample T test (CON *vs.* EXP), and Welch and Games-Howell Tests (different groups) were used. The relationships between changes in performance and changes in coordination were analyzed using the Pearson’s correlation coefficient. All data were processed with SPSS Statistics v.26, and the statistical significance was set at *p* < 0.05.

## Results

A total of 80 subjects completed the RT program, but eight of them missed one or more tests, making the adherence rate of the program 88.9%. There were no adverse events or injuries throughout the study. There were no significant differences in the anthropometry indexes (height, weight, BMI) of the subjects. In the baseline test (Table 2), for those who had only trained for one year, the EXP group had better performance in WB (*p* = 0.030, Cohen’s *d* = 0.755) and 25m freestyle (*p* = 0.009, Cohen’s *d* = 0.921) than the CON group. For those who had trained two years, the EXP group had better performance in WB (*p* = 0.038, Cohen’s *d* = 1.088) and JS (*p* = 0.030, Cohen’s *d* = 1.209) than the CON group. For those who had trained four years, the performance of the EXP group was better in the 25 m freestyle (*p* = 0.000, Cohen’s *d* = 2.127) than the CON group.

**Figure 4:**
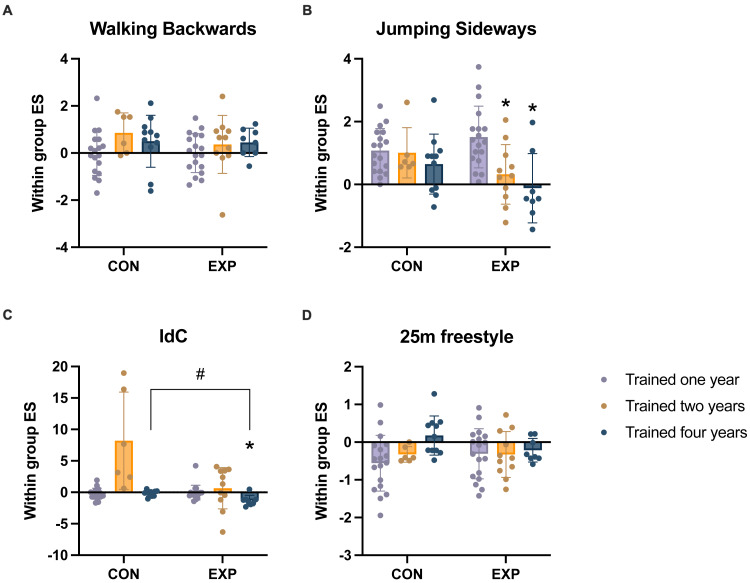
Changes in coordination and swimming performance. (A) Changes in walking backwards performance. (B) Changes in jumping sideways performance. (C) Changes in the index of coordination (IdC). (D) Changes in 25m freestyle performance. The *y*-axis represents within group ES (within group ES = (post-pre)/SD); * *p* < 0.05, compared with one year of training; # *p* < 0.05, compared with CON with same number of training years.

Changes in JS performance was greater in the EXP than in the CON, and there was an interaction between groups and training years (F (2, 66) = 3.60, *p* = 0.033). A subsequent simple effect analysis revealed a significant effect of training years on change in JS performance in EXP(F (2, 34) = 9.20, *p* = 0.001, with partial eta squared of 0.351). There was no significant effect of number of training years on change in JS performance in CON (F (2, 32) = 1.00, *p* = 0.378 with partial eta squared of 0.059). In EXP, the improvement of JS performance in E2 (*p* = 0.014) and E3 (*p* = 0.002) was lower than in those with one year of training experience (Fig. 4).

The changes of the IdC were affected by number of training years in EXP (*F*_Welch_ (2, 17.92) =5.48, *p* = 0.014), and improvements in the IdC were significantly larger for E3 than E1 (*p* = 0.015). For athletes with four years of training experience, improvements in the IdC were significantly larger in EXP than in CON (*p* = 0.001, Cohen’s *d* = 1.718; Fig. 4).

**Figure 5:**
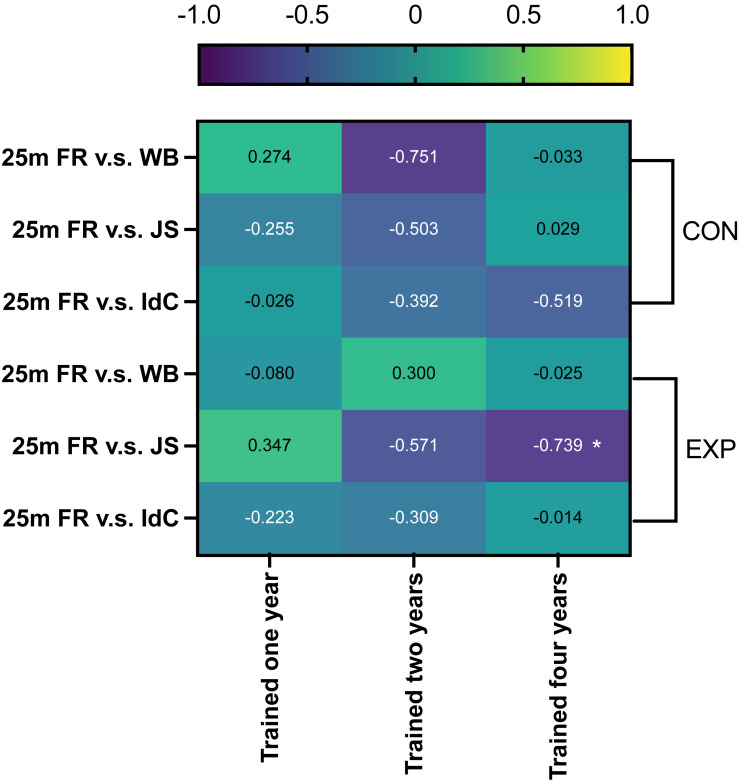
Correlations between changes in swimming and coordination performance in different groups. The change in performance is represented as within group ES (within group ES = (mean post-mean pre)/SD); 25m FR = 25m freestyle; WB = walking backwards; JS = jumping sideways; *significantly correlated (*p* < 0.05).

There was a significant negative correlation between the within group ES in the 25 m freestyle and JS in the EXP swimmers with four years of training experience (Pearson’s *r* =  − 0.739, *p* = 0.036, *r*2 = 0.546), indicating that as the JS improved, 25 m freestyle performance increased (Fig. 5).

## Discussion

Our study showed that RT led to greater improvement in JS performance in young swimmers, and years of sport-specific training experience had a significant impact on JS performance. Specifically, swimmers with two years or four years of training experience showed less improvement in JS performance than those with only one year of training experience. Conversely, rhythm training had no effect on WB performance improvement in all swimmers, regardless of training experience. Overall, our research suggests that general motor coordination can be trained through progressive rhythm training. However, the sensitivity and trainability of rhythm training may vary with the swimming-specific training experience of young swimmers.

The results of research carried out in recent years show that the greatest adaptability of motor coordination abilities occurs between 7 and 12, when athletes are easier to train and the training is most effective (Bompa & Carrera, 2015; Ergash et al., 2020; Gierczuk & Sadowski, 2015; Knjaz, Rupčić & Verunica, 2007). The results obtained in this study align with the conclusions of previous research that the purposeful training can produces a distinct impact on dynamics and improvement rates of different motor coordination abilities in the long-term development of athletes, and that the earlier certain coordination training is introduced, the greater the degree of general motor coordination improvement is likely to be.

It is notable that changes in JS and WB performance after RT differed for swimmers with different years of swim-specific training. There was no significant difference in WB performance changes after rhythm training in swimmers across all years of training experience. Previous studies suggest that WB and JS represent different types of coordination (Jaakkola, Watt & Kalaja, 2017; Nascimento, Henrique & Marques, 2019; Stupar, Romanov & Tomić, 2019): JS tests the ability to coordinate under time pressure while WB tests the ability to coordinate under precision pressure. The results of this study indicate that the RT program used in this study targeted time coordination more than precision coordination. This indicates that in order to help young athletes develop comprehensive coordination skills, training aimed at improving different types of coordination should be done in the early stages of basic motor skill development

The second objective of our study was to assess differences in sport-specific coordination of young swimmers with different training experience after a 12-week RT program. Compared with the CON, the swimmers with more years of training experience in EXP achieved greater improvement in sport-specific coordination after RT.

Rostock and Zimmermann have proposed in their theoretical model of coordination training that there is interdependence between motor coordination abilities and motor skills (Rostock & Zimmermann, 1997). Silva et al. (2019a), Silva et al. (2019b) and Silva et al. (2022) have reported in their studies that there is a high inter-individual variability in stroke coordination in young swimmers. Stroke rate and swimming speed, which are highly correlated with swimming-specific skill performance, showed multiple sensitivities across swimming-specific coordination patterns. In addition to playing an important role in the acquisition and execution of motor skills, motor coordination abilities (*e.g.*, rhythmic ability) are also enhanced as part of the process of learning, training, and practicing sport-specific skills (Hirtz, 1985). Our study indicates that improvements in the JS performance of young swimmers might be logically linked to their swimming-specific training history, meaning that more intensive swimming-specific training experience might also have a promoting effect on the general coordination performance (JS) of young swimmers.

Research on the correlation between motor perception ability and musical rhythm sense shows that the rhythmic stimulus can be used as a neuromodulator between motion perception and rhythm sense (Overy & Turner, 2009). In the movement cycle of swimming, the swimmer must first create “support” with movements that are diagonal to the swimming direction. The event density per unit of time is high, because the temporal coupling of the partial movements of the arms pulling/rolling, legs kicking, and head rotating during inhalation and exhalation occur around the longitudinal axis of the body in about one second. In addition, the joint angles and the angular speeds of these partial movements change several times in the movement cycle (Frank, 2008). This indicates a similarity between swimming-specific training and rhythm training, which together contribute to the improvement of swimming-specific coordination.

The IdC measures coordination between the arms. When the swimmer shows uninterrupted propulsion between both arms, the IdC is 0; this kind of coordination is called *in opposition*. When there is non-propulsive lag time in the arm strokes, the coordination pattern is called *catch-up* and when there are overlap phases in the arm propulsion, the pattern is called *superposition* (Chollet, Chalies & Chatard, 2000). Compared with the catch-up and superposition patterns, the opposition pattern is the most consistent with the characteristics of coordinated motion. Swimmers at lower performance levels usually use a catch-up pattern (Silva et al., 2022). The same observation applies to all swimmers in this study: there were only observed changes in the IdC in the catch-up pattern.

Although this study also found differences in the IdC changes of athletes with different years of training experience in the EXP, it is not clear if these changes were related to the RT. Training experience may have influenced the results because movement variability varies with the development of the central nervous system (Boyer, Freedman Silvernail & Hamill, 2017; Denckla, 1974), showing a decreasing trend from childhood, adolescence to adulthood (MacDonald, Nyberg & Tin Bäckman, 2006). This likely explains why the IdC did not significantly change in E1 and E2, suggesting that the coordination patterns of younger swimmers also depends on the maturation of their central nervous system. There was, however, a significant increase in the improvement of arm coordination in E3, suggesting a decrease in lag time between arm propulsions. This improvement in coordination can be explained by swimming-specific training experience and the maturation of the central nervous system. The RT training program may have affected these improvements, but more evidence is needed to verify that connection.

Another notable result of this study was that in participants with four years of swimming training experience, improvement in general motor coordination was highly associated with improvement in 25 m swimming performance. The regulatory and control system of movement determined the individual’s propensity for coordination, which was expressed externally as economy and accuracy of movement (Bojkowski et al., 2022).

Before swimming-specific performance stabilized, general coordination performance, which reflects the fluidity of movement, also showed a greater degree of dispersion in the E1 and E2 groups. General coordination increases as the athlete’s specialization level further develops because they can rely on the broad technical and motor skills acquired during their early coordination training (D’Isanto et al., 2019). This conclusion has also been confirmed in a study of young tennis players (Waldziński, 2013) which showed that the level of motor coordination developed during the early training process of the athletes was helpful for understanding coordination training sensitivity in motor development and could predict training outcomes at a certain period in an athlete’s career.

One limitation of this study is the uneven distribution of gender in the whole sample and subsample. One of the weaknesses of this study is the incomplete overview of the developmental maturity of the participants. More longitudinal and interventional studies are needed to further investigate the mixed effects of different types of coordination training and sport-oriented practice on the development of coordination abilities during childhood and adolescence. Including samples of athletes from a variety of sports would also further reveal the effect of sport-specific training experience on the development of motor coordination. The influence of gender and biological age factors on the effect of coordination training should also be included in future studies.

## Conclusions

This study adds to the evidence on the effectiveness of early RT in swimmers, showing that interventions designed to address general and sports-specific motor coordination in children and adolescents are feasible in long-term athletic development. Practitioners are encouraged to integrate exercises that address all aspects of motor coordination when developing training programs for youth, especially for children in early childhood, to fully reflect the diversity of motor coordination. Although each sport requires different motor coordination abilities, attention should be paid to the adaptation of young athletes with different levels of sport-specific training experience to the mixed effects of coordination training and sport-specific training.

##  Supplemental Information

10.7717/peerj.15667/supp-1 Supplemental Information 1 Detailed information for the index of coordination calculation

10.7717/peerj.15667/supp-2 Supplemental Information 2 Raw measurement data The original test results for each athlete and the difference between the pre-test and post-test (within group ES).

## Additional Information and Declarations

### Data Availability

The following information was supplied regarding data availability: The raw measurements are available in the Supplemental Files.

## References

[ref-1] Ahnert J, Schneider W, Bös K (2010). Developmental changes and individual stability of motor abilities from the preschool period to young adulthood. Human development from early childhood to early adulthood.

[ref-2] Aly Shady A, Abdelsattar Mahmoud M (2014). Effect of spatial orientation and motor rhythm trainings on motor speed and skill performance level of soccer juniors. Journal of Applied Sports Science.

[ref-3] Bojkowski Ł, Kalinowski P, Śliwowski R, Tomczak M (2022). The importance of selected coordination motor skills for an individual football player’s effectiveness in a game. International Journal of Environmental Research and Public Health.

[ref-4] Bompa T, Carrera M (2015). Conditioning young athletes.

[ref-5] Bös K, Schlenker L (2011). Deutscher Motorik-Test 6–18 (DMT 6–18). Bildung im Sport.

[ref-6] Boyer KA, Freedman Silvernail J, Hamill J (2017). Age and sex influences on running mechanics and coordination variability. Journal of Sports Sciences.

[ref-7] Chollet D, Chalies S, Chatard J (2000). A new index of coordination for the crawl: description and usefulness. International Journal of Sports Medicine.

[ref-8] Dekerle J (2020). High performance youth swimming.

[ref-9] Denckla MB (1974). Development of motor co-ordination in normal children. Developmental Medicine & Child Neurology.

[ref-10] D’Isanto T, D’Elia F, Raiola G, Altavilla G (2019). Assessment of sport performance: theoretical aspects and practical indications. Sport Mont.

[ref-11] Ergash N, Kamila K, Kahhor G, Fazliddin K (2020). Development of coordination abilities and balance of primary school age children gulistan state university. European Journal of Molecular & Clinical Medicine.

[ref-12] Figueiredo P, Silva A, Sampaio A, Vilas-Boas JP, Fernandes RJ (2016). Front crawl sprint performance: a cluster analysis of biomechanics, energetics, coordinative, and anthropometric determinants in young swimmers. Motor Control.

[ref-13] Frank G (2008). Koordinative Fähigkeiten im Schwimmen der Schlüssel zur perfekten Technik.

[ref-14] Gierczuk D, Sadowski J (2015). Dynamics of the development of coordination motor abilities in freestyle wrestlers aged 16-20. Archives of Budo.

[ref-15] Golle K, Mechling H, Granacher UJB (2019). Bewegung, training, leistung und gesundheit.

[ref-16] Hirtz P (1985). Koordinative Fähigkeiten im Schulsport: vielseitig—variationsreich—ungewohnt.

[ref-17] Hopkins WG (2002). A scale of magnitudes for effect statistics. A new view of statistics. www.sportsci.org/resource/stats/effectmag.html.

[ref-18] Jaakkola T, Watt A, Kalaja S (2017). Differences in the motor coordination abilities among adolescent gymnasts, swimmers, and ice hockey players. Human Movement.

[ref-19] Jagiełlo W, Kalina RM (2007). Properties of motor development in young judokas. Journal of Human Kinetics.

[ref-20] Jerzy S, Pawel W, Janusz Z, Tomasz N, Mariusz B (2015). Structure of coordination motor abilities in male basketball players at different levels of competition. Polish Journal of Sport and Tourism.

[ref-21] Kiphard EJ, Schilling F (2007). Körperkoordinationstest für kinder: KTK.

[ref-22] Knjaz D, Rupčić T, Verunica Z (2007). Razvoj koordinacije kroz senzitivna razdoblja s posebnim naglaskom na košarkaške programe.

[ref-23] Kröger C, Roth K, Schmidt L (2014). Koordinationsschulung im Kindes-und Jugendalter: Eine Übungssammlung für Sportlehrer und Trainer.

[ref-24] Lohman T, Roche AF, Martorell R (1988). Anthropometric standardization reference manual.

[ref-25] Lyakh V, Jaworski J, Wieczorek T (2007). Genetic endowment of coordination motor abilities a review of family and twin research. Journal of Human Kinetics.

[ref-26] MacDonald SW, Nyberg L, Tin Bäckman LJ (2006). Intra-individual variability in behavior: links to brain structure, neurotransmission and neuronal activity. Trends in Neurosciences.

[ref-27] Magill R, Anderson D (2010). Motor learning and control.

[ref-28] Miura A, Kudo K, Ohtsuki T, Kanehisa H (2011). Coordination modes in sensorimotor synchronization of whole-body movement: a study of street dancers and non-dancers. Human Movement Science.

[ref-29] Nascimento W, Henrique NR, Marques M (2019). KTK motor test: review of the main influencing variables. Revista Paulista de Pediatria.

[ref-30] Neuper A (2015). Koordinationstraining im Nachwuchsfußball. Diplomarbeit. Karl-Franzens-Universität Graz.

[ref-31] Nieber L (2015). Systematisches Koordinationstraining im Nachwuchshandball. Leistungssport.

[ref-32] Oberger J, Romahn N, Opper E, Tittlbach S, Wank V, Woll A, Worth A, Bös K (2006). Untersuchungen zur motorischen Leistungsfähigkeit und körperlich-sportlichen Aktivität im Rahmen des Kinder- und Jugendgesundheitssurveys des Robert Koch-Institutes Berlin. Assessmentverfahren in Gesundheitssport und Bewegungstherapie: Messen, Testen, Beurteilen, Bewerten Jahrestagung der dvs-Kommission Gesundheit, Saarbrücken, vom 23-24 September 2004 Hrsg: G Wydra: Czwalina.

[ref-33] Opstoel K, Pion J, Elferink-Gemser M, Hartman E, Willemse B, Philippaerts R, Visscher C, MJPo Lenoir (2015). Anthropometric characteristics, physical fitness and motor coordination of 9 to 11 year old children participating in a wide range of sports. PLOS ONE.

[ref-34] Overy K, Turner R (2009). The rhythmic brain. Cortex.

[ref-35] Pion JA, Fransen J, Deprez DN, Segers VI, Vaeyens R, Philippaerts RM, Lenoir M (2015). Stature and jumping height are required in female volleyball, but motor coordination is a key factor for future elite success. The Journal of Strength and Conditioning Research.

[ref-36] Platvoet S, Faber IR, De Niet M, Kannekens R, Pion J, Elferink-Gemser MT, Visscher C (2018). Development of a tool to assess fundamental movement skills in applied settings. Frontiers in Education.

[ref-37] Rostock J, Zimmermann K (1997). Koordinationstraining zwischen Generalität und Spezifität. Leistungssport.

[ref-38] Seifert L, Komar J, Barbosa T, Toussaint H, Millet G, Davids K (2014). Coordination pattern variability provides functional adaptations to constraints in swimming performance. Sports Medicine.

[ref-39] Silva AF, Figueiredo P, Morais S, Vilas-Boas JP, Fernandes RJ, Seifert L (2019a). Task constraints and coordination flexibility in young swimmers. Motor Control.

[ref-40] Silva AF, Figueiredo P, Ribeiro J, Alves F, Vilas-Boas JP, Seifert L, Fernandes R (2019b). Integrated analysis of young swimmers’ sprint performance. Motor Control.

[ref-41] Silva AF, Seifert L, Fernandes RJ, Vilas Boas JP, Figueiredo P (2022). Front crawl swimming coordination: a systematic review. Sports Biomechanics.

[ref-42] Söğüt M, Kirazci S, Korkusuz F (2012). The effects of rhythm training on tennis performance. Journal of Human Kinetics.

[ref-43] Stupar D, Romanov R, Tomić B (2019). KTK test for assessment of coordination of young karatist. TIMS Acta.

[ref-44] Vandorpe B, Vandendriessche J, Lefèvre J, Pion J, Vaeyens R, Matthys S, Philippaerts R, Lenoir M (2011). The Körperkoordinationstest für kinder: Reference values and suitability for 6–12-year-old children in Flanders. Scandinavian Journal of Medicine & Science in Sports.

[ref-45] Vandorpe B, Vandendriessche JB, Vaeyens R, Pion J, Lefevre J, Philippaerts RM, Lenoir M (2012). The value of a non-sport-specific motor test battery in predicting performance in young female gymnasts. Journal of Sports Sciences.

[ref-46] Waldziński T (2013). Coordination motor abilities of the boys aged 9–12 training tennis.

[ref-47] Weineck J (1996). Training manual.

[ref-48] Weineck J, Memmert D, Uhing M, Memmert D (2012). Optimales Koordinationstraining im Fußball: Sportwissenschaftliche Grundlagen und praktische Umsetzung.

[ref-49] Zahradník D, Korvas P (2017). The introduction into sports training.

