# Supplementary material for: The effect of rhythm training on the motor coordination abilities of 8–12-year-old freestyle swimmers
Source: PeerJ. 2023 Jul 28;11:e15667. doi: 10.7717/peerj.15667 (PMC10389069; doi:10.7717/peerj.15667)
Supplement: Supplemental Information 1 [file peerj-11-15667-s001.pdf]

Supplemental Article

The mean duration (T) of a complete arm movement was defined as the sum of the phases (T1 + T2 + T3 + T14). Within the equation, T1 refers to the duration of Phase A(entry and catch of the hand in the water), T2 refers to the duration of Phase B (pull), T3 refers to the duration of Phase C (push), and T4 refers to the duration of Phase D (recovery) (Chollet, Chalies et al. 2000).

$$T=T1+T2+T3+T4 \cdots\cdots\cdots\cdots \quad (E1)$$

The lag time (LT) corresponded to the time between the beginning of propulsion in the first right-arm stroke and the end of propulsion in the first left-arm stroke (LT1) and between the beginning of propulsion in the second left-arm stroke and the end of propulsion in the first right arm stroke (LT2).

$$LT=\frac{(LT1+LT2)}{2} \cdots\cdots\cdots\cdots (E2)$$

The index of coordination (IdC) was expressed as a percentage of the mean duration (T) of the lag time (LT):

$$IdC =\frac{LT}{T}\% \cdots\cdots\cdots\cdots (E3)$$

Chollet, D., et al. (2000). "A new index of coordination for the crawl: description and usefulness." International Journal of Sports Medicine 21(01): 54-59.
